# Structure/Function Analysis of Cotton-Based Peptide-Cellulose Conjugates: Spatiotemporal/Kinetic Assessment of Protease Aerogels Compared to Nanocrystalline and Paper Cellulose

**DOI:** 10.3390/ijms19030840

**Published:** 2018-03-13

**Authors:** J. Vincent Edwards, Krystal Fontenot, Falk Liebner, Nicole Doyle nee Pircher, Alfred D. French, Brian D. Condon

**Affiliations:** 1 Southern Regional Research Center, USDA, New Orleans, LA 70124, USA; krystal.fontenot@ars.usda.gov (K.F.); Al.French@ars.usda.gov (A.D.F.); brian.condon@ars.usda.gov (B.D.C.); 2 University of Natural Resources and Life Sciences Vienna, Konrad-Lorenz-Straße 24, A-3430 Tulln an der Donau, Austria; falk.liebner@boku.ac.at (F.L.); nicole.pircher@boku.ac.at (N.D.n.P.)

**Keywords:** nanocellulose, protease sensor, human neutrophil elastase, peptide-cellulose conformation, aerogel

## Abstract

Nanocellulose has high specific surface area, hydration properties, and ease of derivatization to prepare protease sensors. A Human Neutrophil Elastase sensor designed with a nanocellulose aerogel transducer surface derived from cotton is compared with cotton filter paper, and nanocrystalline cellulose versions of the sensor. X-ray crystallography was employed along with Michaelis–Menten enzyme kinetics, and circular dichroism to contrast the structure/function relations of the peptide-cellulose conjugate conformation to enzyme/substrate binding and turnover rates. The nanocellulosic aerogel was found to have a cellulose II structure. The spatiotemporal relation of crystallite surface to peptide-cellulose conformation is discussed in light of observed enzyme kinetics. A higher substrate binding affinity (*K*_m_) of elastase was observed with the nanocellulose aerogel and nanocrystalline peptide-cellulose conjugates than with the solution-based elastase substrate. An increased *K*_m_ observed for the nanocellulosic aerogel sensor yields a higher enzyme efficiency (*k*_cat_/*K*_m_), attributable to binding of the serine protease to the negatively charged cellulose surface. The effect of crystallite size and β-turn peptide conformation are related to the peptide-cellulose kinetics. Models demonstrating the orientation of cellulose to peptide O6-hydroxymethyl rotamers of the conjugates at the surface of the cellulose crystal suggest the relative accessibility of the peptide-cellulose conjugates for enzyme active site binding.

## 1. Introduction

### Nanocellulose-Based Protease Sensors

The sophistication and sensitivity of point of care diagnostic detectors underlies much of the progress in the rapidly growing field of biosensors as is found with point of care protease sensors [1]. The sensor’s transducer serves to ground and facilitate transformation of the sensor molecule (in this study, a peptide fluorophore) into a signature signal, which in this study is a fluorescence response indicating proteolytic activity. However, the composition of the transducer surface material and its interaction at the biomolecular interface (protease binding on the transducer surface) is of equal value. Another important consideration is the detectors interface with the biological environment, which can complicate and dampen the sensitivity of detection. This is especially the case when considering the design of in situ protease sensors interfaced with dressings to detect harmful levels of proteolytic enzymes.

Proteolytic enzymes including, matrix metalloproteases (MMPs) and serine proteases such as the neutrophil serine protease (human neutrophil elastase, HNE), are responsible for proteolytic degradation of growth factors [2] and the extracellular matrix (ECM) proteins in chronic wounds. In general, acute wounds have normal levels of MMPs and HNE [3,4,5], which facilitates the clearance of cellular debris. However, chronic wounds have elevated levels of MMPs (0.1–0.2 U/mL) and HNE (0.02–0.1 U/mL) depending on the type, e.g., diabetic, venous, pressure, and arterial ulcers [5]. Thus, elevated concentrations of MMPs and HNE delay the healing process, and are also considered biomarkers for chronic wound treatment evaluation [6,7,8]. 

Biosensor materials should be compatible with the complexity of the bio-system they are applied to. This is especially relevant to the chronic wound environment, which varies in exudative fluid levels, bacterial burden, and biochemical diversity. Biocompatibility is imparted to resist nonselective surface adsorption by lipids, proteins, polysaccharides, cellular debris, and breakdown of the transducer surface. Due to cellulosic’s hydrophilic properties, protein binding on the transducer surface for sensor detection is relatively attenuated when compared with more hydrophobic materials. 

We have recently examined the relative contributions of cellulosic and nanocellulosic sensor transducer surfaces to detection sensitivity of the neutrophil protease, HNE, demonstrating that different forms of cellulose and nanocellulose materials have varying surface area, porosity, and biocompatible properties affording sensor/transducer surface functionality [9,10,11,12,13,14]. 

**Figure 1:**
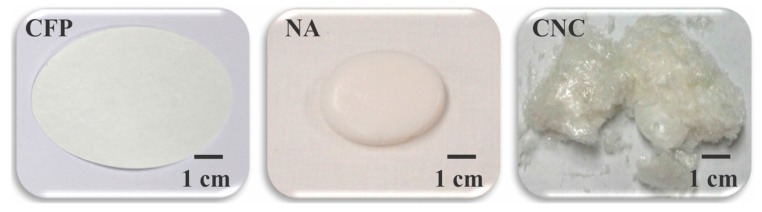
Images of the cellulosic (CFP) and nanocellulosic (NA and CNC) transducers.

Notably it is worth considering that nanocellulose is a highly crystalline biopolymer with a hydrophilic and high specific surface area that possesses reactive hydroxyls, which can be derivatized to covalently append a wide range of biologically active molecules. Here, the focus is on comparison of the relative structure/function contributions of three different cotton transducer surfaces (nanocellulose crystals (CNC), nanocellulosic aerogels (NA), and cellulose filter paper (CFP) (Figure 1), each having unique properties contributing to protease sensor sensitivity, yet varying features of sensor and dressing applicability. The sensor molecule is a tripeptide substrate of elastase. Accordingly, we examine here a nanocellulosic aerogel material in comparison with two other cellulosic and nanocellulosic designs for protease-lowering activity, and sensor/transducer surface performance with the aim of understanding how the molecular and physical properties of the materials may provide benefit for a dressing/sensor platform useful to simultaneously test and treat targeted elevated protease levels in chronic wounds.

In this study, we evaluate the relative structure/function contributions of the protease substrate, which is immobilized to a nanocellulosic aerogel. The structure function approach analyzes the enzyme kinetic turnover rate and binding affinity of the enzyme to structural findings of the crystal structure that influences the immobilized peptide in a β turn conformation on the cellulose crystallite surface in its interaction with the protease. Accordingly, the structure/function relations of two nanocellulosic and a cellulosic sensor analogs are contrasted, and also demonstrate interesting differences in protease enzymology. 

## 2. Results

### 2.1. Structure/Function and Physical Property Considerations

A depiction of the physical structure of the materials of this study is shown in Figure 1. The intended application of the nanocellulose aerogel sensor is as an interface with chronic wound dressings to detect protease levels in situ. The functional properties of a protease sensor are determined by the composition of the transducer surface, the bioactivity of the enzyme substrate attached, and how the substrate is immobilized. A cellulose-based transducer surface, to which the peptide protease substrate is covalently attached, may be characterized for its crystalline structure and physical property composition [13,15]. In this study, conjugation of the tripeptide elastase substrate (Suc-Ala-Pro-Ala-AMC) to the cellulosic and nanocellulosic transducers via the amide bond formation between the carboxyl terminus of the tripeptide and the reactive hydroxyls on the transducer surfaces afforded the peptide-cellulose conjugates, pCFP, pNA and pCNC, which has previously been outlined [9,12].

### 2.2. Structure/Function Relations of the Protease Activity at a Molecular Level

#### 2.2.1. Cellulose Crystal Structure

X-ray crystallography of the different transducer surfaces was undertaken and used to compare the relative contributions of cellulose structure to the peptide-cellulose conjugates and structural relationships of the cellulosic transducer surfaces to enzyme kinetics.

**Figure 2:**
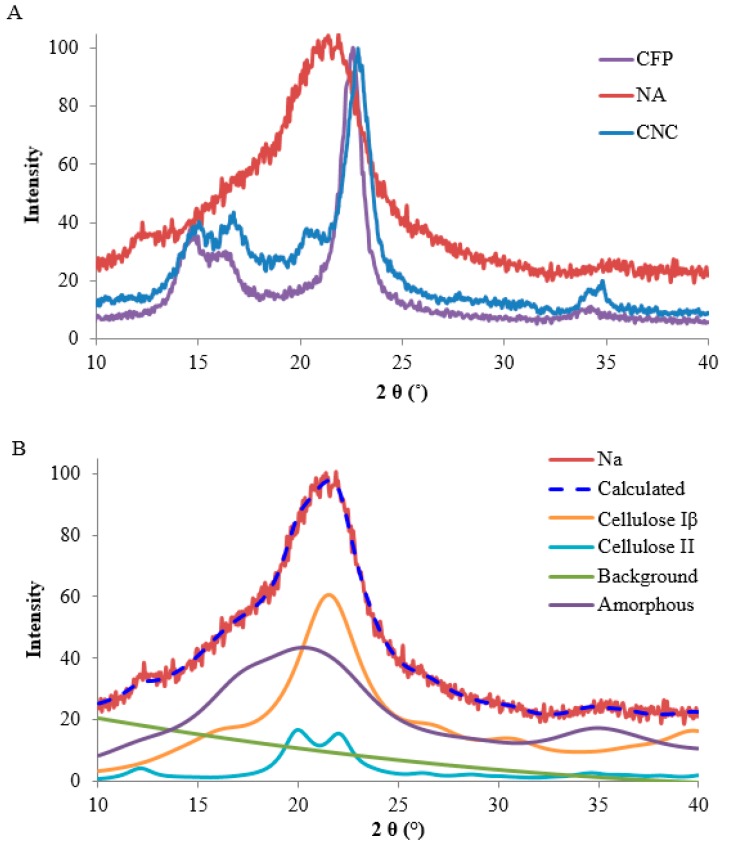
X-ray observed diffraction patterns (**A**) of the CFP, NA, and NC transducers; and (**B**) the calculated pattern of the NA as analyzed using the Rietveld method.

Figure 2 shows patterns from the X-ray diffraction (XRD) analyses. In the case of the CFP and the CNC, which are cotton based materials, the patterns were typical cellulose I_β_ patterns. The pattern from the aerogel transducer, seemingly a mostly amorphous material, is also shown in Figure 2A. That pattern was subjected to Rietveld analyses to do what was reasonable to confirm the initial diagnosis of amorphous material. The results of the Rietveld analysis are presented in Figure 2B.

Table 1 shows the larger values of Segal CrI (Crystallinity Index) and crystallite size for the CFP, compared to CNC. Apparently, the acid hydrolysis of the cotton reduced somewhat the size of the crystallites.

A more careful visual analysis of the NA pattern indicates a mixture of cellulose I, II and amorphous structures. [16]. The indications are the small peak at about 12° 2θ (indicating II) as well as the convex intensity in the area of 15°–17° 2θ which indicated some contributions from cellulose I. The presence of some crystalline material was supported by the poor fit of a calculated pattern from very small cellulose II crystals. Earlier, Nam et al. [17] found that such patterns with a very broad peak closely resembled exhaustively ball-milled cotton. Contributions of calculated patterns from a small cellulose I crystal improved the fit, as did one from a relatively large cellulose II crystal. Ultimately, a close fit was obtained between the calculated and experimental patterns, but it was necessary to include a substantial contribution from preferred orientation to obtain the fit depicted in Figure 2B. Although the analysis indicated 44% of the small cellulose I crystallites, 48% of amorphous as modeled by small cellulose II crystallites, and 8% of large crystallites of II, these values should be taken as indications of the presence of three phases, not precise values. In addition, it is notable that crystallinity index values, i.e., Segal CrI (shown in Table 1) for CFP and CNC, are consistent with previously determined values for filter paper and cotton nanocrystalline cellulose. Such analyses are difficult; to get a visually satisfactory fit, 17 variables were used in the refinement with the most severe being the March–Dollase compensation for preferred orientation applied to the cellulose I component. This distorted the cellulose I pattern in Figure 2A. Because of the limited, somewhat noisy data, the obtained fit is not likely to be unique; other models could also give plausible analyses. 

It is noted that these values for the composition were calculated from the exported data from the MAUD (materials analysis using diffraction) Rietveld software, not the values reported during the analysis. However, indications available are that there is considerably more surface area available for the NA samples than for the CFP and CNC materials because of the mostly smaller crystallites of NA.

The presence of the cellulose II crystallites is in good agreement with a previous study comparing the nanomorphology of various types of cellulose II aerogels obtained by dissolution-coagulation of cotton linters using different cellulose solvent systems. For calcium thiocyanate octahydrate/lithium chloride, i.e., the solvent used to prepare the three-dimensional NA scaffolds in this study, small angle X-ray spectroscopy had revealed considerably high cellulose II crystallinity (ca. 50%) and a crystal diameter of 46 ± 1 Ǻ [18]. Notably, the crystallite size impacts the sensitivity of the sensor by increasing the specific surface area of the sensor which is correlated with sensor sensitivity [12].

**Figure 3:**
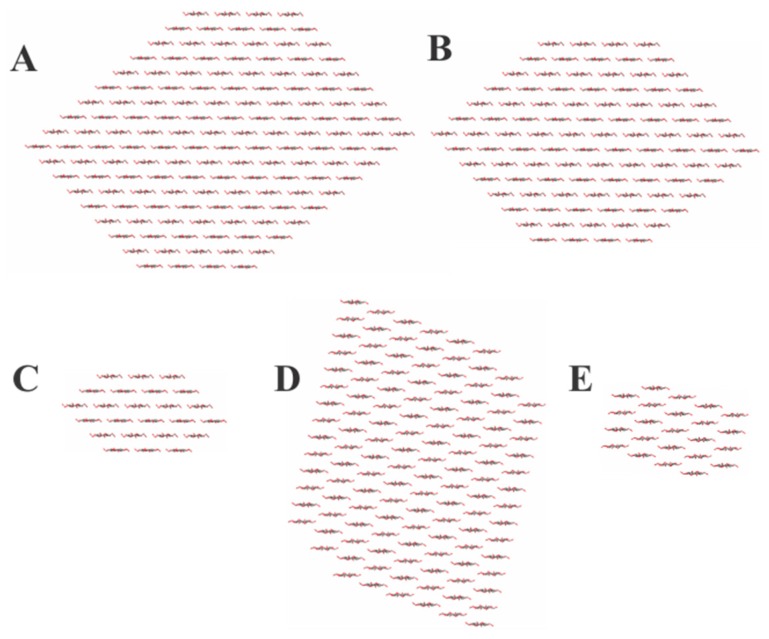
Models of the cotton cellulose and nanocellulose transducer materials based on the X-ray diffraction patterns. (**A**) CFP; (**B**) CNC; and (**C**–**E**) cellulose I, cellulose II, and amorphous contributions to the diffraction pattern for NA.

The crystallite models (shown in Figure 3) that are based on the respective cellulose crystallite sizes reveal the relative number of cellulose layers and chains which are CFP (18 layers and 144 chains), NA (12 layers and 78 chains), and CNC (14 layers and 98 chains). The number of chains (36, 28, and 24) on the 1–10 and 110 surfaces for the CFP, CNC, and NA, respectively, indicate half of the primary alcohol groups on those surface chains are in the interior structure of the cellulose or nanocellulose materials [11]. As discussed above, this structural feature effects the available primary hydroxyls for covalent attachment of the sensor molecule.

#### 2.2.2. Peptide Conformation Consideration Based on Circular Dichroism

**Figure 4:**
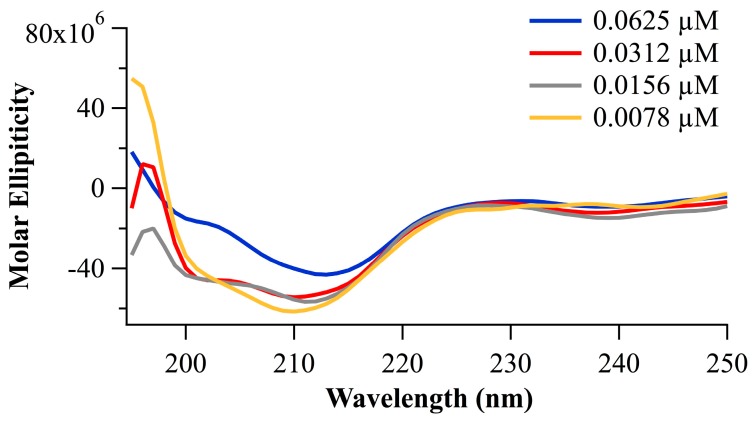
Circular Dichroism of the tripeptide (Suc-APA-AMC) at various concentrations ranging from 0.0625 to 0.0078 μM.

Here, we provide experimental evidence for secondary structure in the peptide portion of the conjugate. As shown in Figure 4, circular dichroism spectra of the peptide portion of the conjugate, i.e., Suc-Ala-Pro-Ala-AMC, are consistent with a β-turn prediction. Reflected in the CD are spectral minima at 200 (lit 208) and 214 (lit 222) nm, which are typical for alpha helices, indicate a propensity for type I β-turn formation [19], and with strong evidence for the type I β-turn formation as previously characterized for Pro-Ala [20].

#### 2.2.3. Bioactivity and Kinetic Evaluation of Peptide-Cellulose Conjugates with Elastase

**Figure 5:**
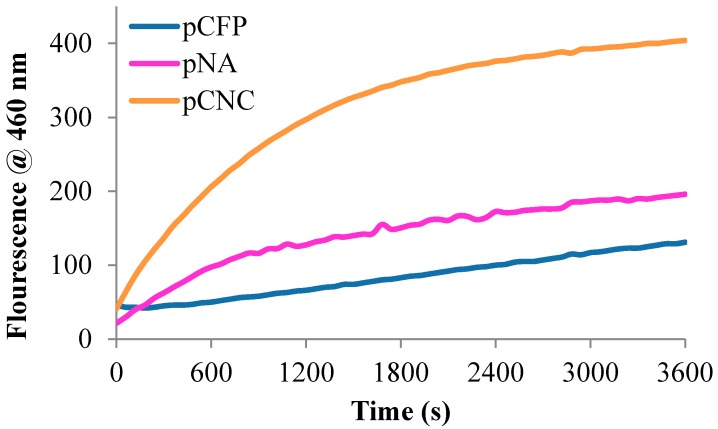
The response curves of 2 mg of pCFP (blue), pNA (pink), and pCNC (green) biosensor upon detection of 7-amino-4-methylcoumarin released with HNE at 0.5 U/mL substrate hydrolysis at 37 °C.

Here, the sensor’s ability to detect protease activity and the sensor functionality itself is determined through protease assays typically used to assess the enzyme/substrate recognition and relevant substrate binding and enzyme kinetics. In this study, it is applied to immobilized enzyme substrates i.e., the peptide-cellulose conjugates. The elastase activities of the tripeptide-conjugated sensor analogs, were assessed with succinamidyl-Ala-Pro-Ala-amidylcoumadin peptide-cellulose ester immobilized on the transducer surfaces through a synthesis that has previously been reported [11,13]. The bioactive response was assessed through fluorescence resulting from the protease catalyzed release of the COOH-terminal amino methyl coumarin fluorophore. The results of the bioactivity are shown in Figure 5 by way of reaction progress curves of each sensor’s reaction with elastase at a protease concentration of 0.5 U/mL HNE. The relative response curves and detection sensitivity are apparent from the reaction progress and sensitivity curves.

The relative kinetic rates of the pCFP, pNA, and pCNC biosensors with HNE was assessed using traditional Michaelis–Menten kinetic analysis. Table 2 lists the kinetic values for the HNE peptide-cellulose substrates as attached in pCFP, pNA, and pCNC. The kinetic values refer to both the binding affinity of the conjugated enzyme substrate and the turnover rate or rate of product formation; a measurement of the rate of formation of hydrolyzed fluorophore (AMC) from the COOH-terminus of the bound peptide. 

## 3. Discussion

### 3.1. Structural Features of Peptide-Cellulose Conjugate

**Figure 6:**
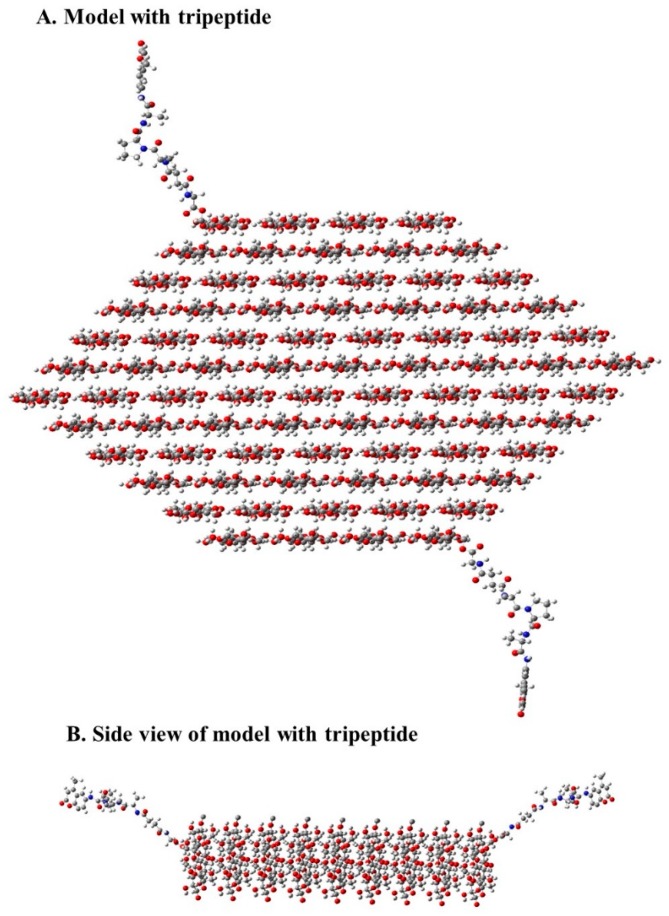
Models of pNA on the X-ray diffraction patterns and Rietveld calculated pattern. The pNA peptide model (**A**) Peptide-cellulose conjugate of aerogel sensor modeled in a single crystal structure with a width of 58.5 Ǻ (78 chains and 12 layers). The side view (**B**) is an image of the same model as in A, but along the longer perimeter of the model.

Figure 6 shows a model of the crystallite-based structure of the peptide cellulose conjugate representative of the ones reported here. To contrast the properties of the nanocellulosic aerogel-based sensor, we include two other cellulose-based sensors that have similarities and differences. Each of the three forms of cellulose and nanocellulose materials evaluated serves as a transducer surface of the protease sensor. Thus, assessment of the relative transducer surface properties that enable sensor protease detection sensitivity was undertaken in the context of contrasting the nanocellulose aerogel-based sensor with peptide-cellulose conjugate analogs of two other distinctly different cellulose transducer surfaces, i.e., porous cotton-based cellulose filter paper substrate (CFP) and cotton nanocellulose crystals (CNC).

As discussed above, during the generation of the NA, a portion of the cellulose is converted to cellulose II [21]. In agreement with earlier reports of several authors [22], we found that different from all other common cellulose solvent systems, cellulose bodies coagulated from respective solutions in calcium thiocyanate octahydrate suffered from distinctly less shrinkage when subjected to solvent exchange and/or scCO_2_ drying. This can be explained by a remaining fraction of non-dissolved cellulose I acting as templating and reinforcing scaffold for coagulated cellulose II in the sense of an all-cellulose composite aerogel [23]. It is also worth noting that cellulose II hydrate has been shown to have a larger unit cell than the dehydrated form [24], which is relevant to a further observation here on the influence of nanocellulose aerogel swelling on detection sensitivity and dressing properties.

The crystallite models (Figure 3) which are based on the respective cellulose crystallite sizes reveal the relative number of cellulose layers and chains which are CFP (18 layers and 144 chains), NA (12 layers and 78 chains), and CNC (14 layers and 98 chains). The number of chains (36, 28, and 24) on the 1–10 and 110 surfaces for the CFP, CNC, and NA, respectively indicate half of the primary alcohol groups on those surface chains are in the interior structure of the cellulose or nanocellulose materials [11]. As discussed above, this effects the available primary hydroxyls for covalent attachment of the sensor molecule. 

Previously, we have discussed the role of the elastase substrate’s peptide secondary structural conformation relative to the peptide-nanocellulosic conjugate’s interaction with the protease from a computational perspective [11] and the potential interaction of the peptide with the crystallite surface. In this regard, the amino acid sequence of this study, Ala-Pro-Ala, has been shown to favor a β-turn conformation [25], which is the smallest element of secondary conformation possible for a protein or peptide. It is also worth noting that β-turns tend to form in more hydrophobic and proteinaceous environments. Thus, the formation of a β-turn in the conjugates of this study may be further enabled by the anionic transducer surface, and upon binding with the protease, which also has a high affinity for the transducer surface, and is likely to populate the transducer surface by way of its positive charge.

### 3.2. Sensor Bioactivity and Kinetics

The bioactivity of the sensor is dependent on the protease’s affinity and interaction with the immobilized substrate and its conjugate surface, which in this study is principally crystalline cellulose. However, we have previously characterized the property of HNE binding to its immobilized substrate from a perspective of adsorption isotherms of elastase binding to the immobilized peptide substrate [26] on crosslinked gels. An assessment of elastase-substrate kinetics and Langmuir adsorption isotherms demonstrated that the protein adsorption at the solid–liquid interface of substrate-bound ethoxyacrylate gels was relatively low i.e., the ratio of maximal adsorbed enzyme to bound ligand was <1%. Thus, in this study the transducer surfaces are negatively charged, and adsorb the HNE marker protein (a positively charged protein) on their crystallite surfaces by way of ionic binding. Accordingly, the negative charge of the transducer surfaces of this study are listed in Table 1, and vary from −10 to −68 mv in order of increasing specific surface area. Moreover, the enhanced HNE binding that these types of transducer surfaces afford is reflected in the Michaelis-Menten kinetics for the immobilized elastase substrate. 

An understanding of the relative peptide conformation and crystal structure of the sensor’s transducer surface as presented above gives a framework in which understanding enzyme binding and the turnover rates of the cellulose peptide conjugate is possible. Moreover, a perspective of the kinetic turnover and binding affinity between the enzyme and the peptide-cellulose analogs, which possess the enzyme substrate, lends itself to an understanding of the mechanism of action of the sensor with the enzyme (HNE).

The 1.5-fold higher *k*_cat_ values observed for the peptide substrate in solution compared with the pNA conjugate reflects a generally slower rate of product formation when the enzyme substrates are attached to the nanocellulosic aerogel. This is understandable in light of the two-phase reaction (due to the insolubility of cellulose and nanocellulose in the assay solution) that is occurring between elastase and the peptide bound to the transducer surface, and is also observed for pCFP and pCNC. The lower V_max_ value, which is the maximum reaction rate mediated by the enzyme, observed for the pNA conjugate is consistent with the enzyme turnover rate decreasing in the conjugates. 

On the other hand, the enzyme-substrate affinity or ability of the substrate to bind to the enzyme active site, as reflected in the *K*_m_ values, was approximately 4-fold greater with the pNA than in solution. Thus, the higher HNE affinity for the tripeptide substrate pNA also gives rise to a higher enzyme efficiency as seen by the higher *k*_cat_/*K*_m_ assigned to pNA; it is 2.5-fold higher than the *k*_cat_/*K*_m_ for the analogous enzyme substrate assessed in solution. These increases in binding can be understood in light of the crystallite size, specific surface area, and negative charge, as discussed above.

As shown previously, the macropores of the paper sensor allow passage of the serine protease to the tripeptide substrate attached within the interior structure whereas mesopores as found in the nanocellulose aerogel would not allow passage of the protease or facilitate reaction with the peptide ligand which has a polar surface area of 2 nm^2^ (note the peptide substrate may be covalently bonded within the nanocellulose aerogel interior due to its relatively small size) [12]. Thus, access of HNE to the interior of the nanocellulose aerogel may be restricted especially since the pore size of pNA is 30 times less than the polar surface area of elastase (309 nm^2^) based on considerations of the material’s structure under non-aqueous condition. This may explain the relatively low binding affinity as measured by *K*_m_ of the nanocellulose aerogel compared with the paper and nanocrystal. Nonetheless, the turnover rate on the nanocellulose aerogel exceeded that of the paper and nanocrystal. This suggests that increasing the pore size of the aerogel to a volume sufficient for passage of the HNE may result in an increase in the overall enzyme efficiency.

### 3.3. Spatiotemporal Considerations of the Peptide-Cellulose Conjugate/Elastase Interaction

Spatiotemporal relationships of the peptide-cellulose conjugate to the protease are influenced by charge, protein and peptide-cellulose conjugate structure, and how these components of sensor structure and function come together in time and space. Thus, based on the kinetic properties of the sensors as discussed above it can be inferred that there are strong molecular interactions between elastase and the peptide cellulose conjugates. Specific molecular influences on sensor function are determined by the β turn peptide conformation, the frequency and orientation of peptide-cellulose conjugates on the crystallite surfaces, and the magnitude of the negative charge on the transducer surface. 

The negatively charged cellulose surface would favor binding to elastase at a region of the protein where a positively charged cluster occurs and is predominated by arginine residues of HNE. Part of this strongly cationic cluster borders on the S1 active site of the serine protease [27]. Thus, it is feasible that the active site of elastase is brought into close proximity of the cellulose surface through charge transfer interactions between the protein and crystallite surface. 

Since the β-turn peptide conformation and its specific resident peptide sequence is required for optimal binding in the oxyanion hole of the active site of the serine protease, stabilization of the conjugate by a negatively charged more hydrophobic surface may increase the enzyme active site binding and in part play a role for the improved *K*_m_ values observed for the nanocellulose aerogel as well as paper and nanocrystal forms when compared with the *K*_m_ determined in the solution phase.

**Figure 7:**
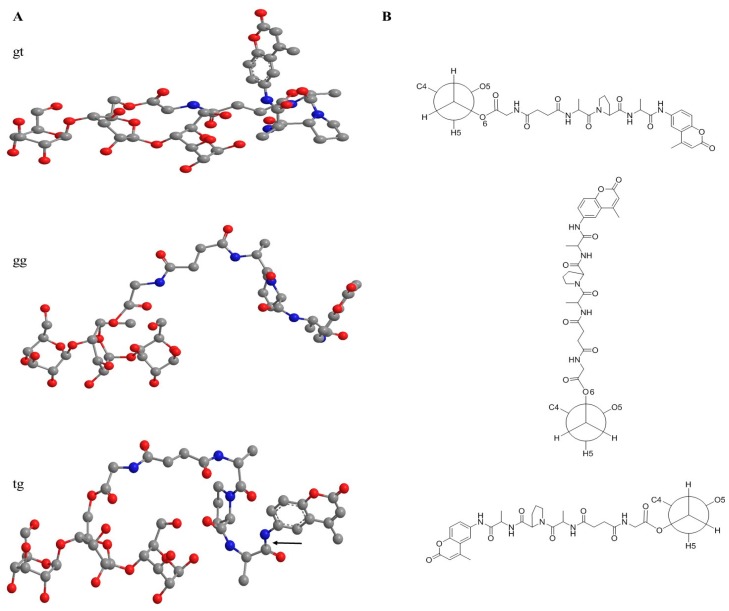
The conformational structures of the tripeptide substrate anchored onto a cellotriose unit at the hydroxymethyl gt O6 rotamer, gg O6 rotamer, and tg O6 rotamer orientation of the cellulose or nanocellulose sensors in: (**A**) minimized model; or (**B**) Newman projections. The black arrow shown for the tg O6 rotamer shows where the HNE protease cleaves the fluorophore. Note: gray is carbon, blue is nitrogen, red is oxygen.

On the other hand, it is important to recognize that the two different structures of cellulose I and cellulose II also play a role in the sensor activity by virtue of the orientation they confer to the conjugate peptide at the O6 rotamer of cellulose’s hydroxymethyl, which is where the peptide is linked to the cellulose. The O6 hydroxymethyl rotamer influences the direction and orientation of the peptide with respect to the cellulose crystallite floor as depicted in Figure 7. For example the conformation of hydroxymethyls in cellulose II has been shown to be predominantly gauche/trans (gt O6) (rotamer oriented from the O5 oxygen through the C4 carbon of anyhydroglucose residues), and conversely the tg O6 rotamer predominates in cellulose I [28]. However, it is worth noting that far less work has been done on cellulose II nanocrystal shapes when they are involved in aerogels compared with cellulose I, and as discussed earlier the coagulation phase of the nanocellulose aerogel preparation may present a unique juxtaposition of minor cellulose polymorphs. Thus, it is likely that the peptide attaches to a gg O6 in both cellulose I and II since the O6 rotamer of the conjugate is not tethered by a hydrogen bond as are the hydroxymethyls in the cellulose crystalline lattice. This is consistent with our examination of a model of a cellulose II crystal where it appears that the top and bottom surfaces have the gt O6 atoms directed to the interior of the crystallite, and may not be favored for the peptide to attach to due to steric hindrance. Accordingly, the gg O6 hydroxymethyl rotamer would orient the peptide outward to the plane of the cellulose crystallite. Thus, we present a minimized model of the peptide cellulose analog in Figure 7 at a hydroxymethyl gg O6 rotamer orientation. 

**Figure 8:**
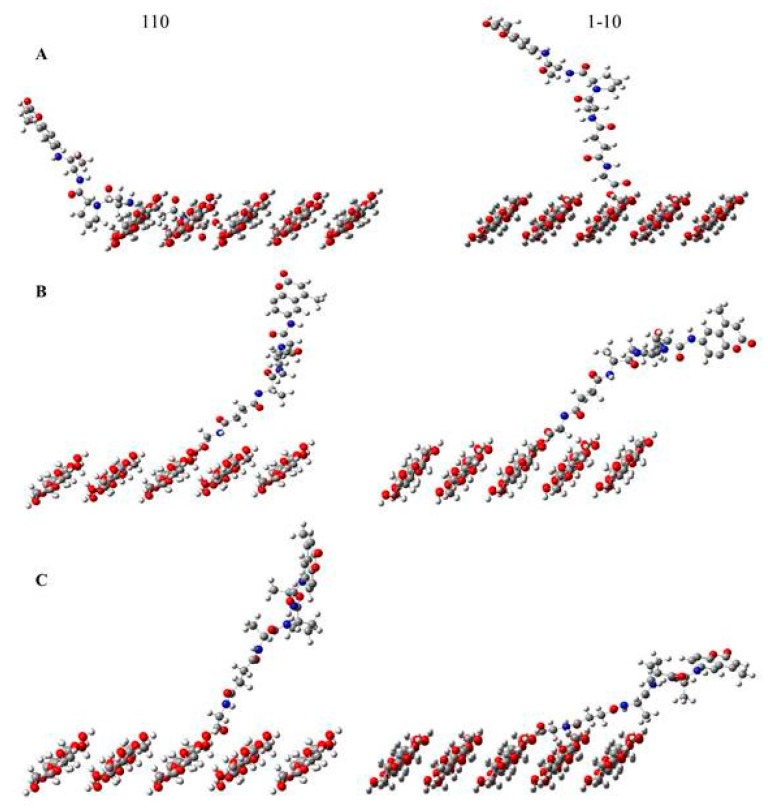
The tripeptide substrate anchored on the five-chain 110 or 1–10 surface of cellulose in the following rotamer positions: (**A**) gauche-gauche; (**B**) gauche-trans; and (**C**) trans-gauche.

To further examine the relationship of the peptides orientation to the crystal surface the different possible combinations of the peptide-cellulose relation to the crystal surface are shown in Figure 8 and the relative distances of the scissile bond of the sensor peptide (the bond that HNE hydrolyzes upon binding and recognition of the enzyme active site) from the cellulose surface plane are listed in Table 3. The 1–10 and 110 surface planes form two sides of the cellulose crystal in both cellulose I and II. As shown the gg O6 rotamer of the 110 surface plane is considerably closer to the surface than found in the 1–10 surface plane. The orientation of hydroxymethyls in the 110 and 1–10 surface plane is predominantly equatorial [29]. In the case of the gt and tg O6 rotamers the proximity of the peptide to the surface planes is reversed to that observed for the gg O6 rotamer. Nonetheless the gg O6 rotamer on the 110 surface plane of the aerogel may account for the lower binding affinity due to increased steric hindrance to binding of the gg O6 110 surface plane rotamer. However, based on the distances measured for all of the combinations modeled on both surface planes it appears that there is a fairly even variation in the distribution of peptide orientations with respect to the crystal surface, and neither surface the 110 or 1–10 surface would predominately influence enzyme turnover from the standpoint of peptide orientation. On the other hand, the 110 surface plane is more hydrophobic due to exposure of fewer hydroxyls on its surface. Thus, the contribution of this feature to enzyme binding and turnover is not clear other than to point out that elastase binding tends to favor more hydrophobic substrates, and it is likely that the relative polarity and charge of the surfaces and the proximity of the peptide together have a pronounced influence on the peptide-cellulose affinity and turnover of the enzyme.

## 4. Materials and Methods

### 4.1. General

Potassium chloride (KCl), sodium chloride (NaCl), hydrochloric acid (HCl), monosodium phosphate (NaH_2_PO_4_), trifluoroethanol (TFE), and sodium hydroxide (NaOH) were purchased from VWR (Radnor, PA, USA). Promogran Prisma^TM^ (Systagenix, San Antiono, TX, USA) was purchased from the distributor HighHealthTide. Human neutrophil elastase (HNE) was purchased as a salt-free lyophilized solid from Athens Research and Technology (Athens, GA, USA) without further purification. The peptide n-succinyl-Ala-Pro-Ala-7-amino-4-methylcoumarin (Suc-Ala-Pro-Ala-AMC) was purchased from BACHEM (Torrance, CA, USA) without further purification. 

### 4.2. Synthesis of Nanocellulosic Aerogel and Generation of Biosensors

The nanocellulosic aerogel was prepared and characterized as previously reported by Edwards et al. [12]. The esterification, and peptide immobilization of the cellulosic print cloth (CPC), cotton nanocellulosic crystals (CNC), and nanocellulosic aerogel (NA) were previously reported by Edwards et al. [9,12]. In summary, glycine-esterified cellulose and nanocellulose substrates were prepared by reacting the glycine α–amino functionality with the COOH-terminal group of Suc-Ala-Pro-Ala-AMC by way of a carbodiimide-mediated reaction. Following a simple work-up of rinsing and drying small representative samples of the active peptide component biosensors were cleaved from the matrices by adding a mixture of TFA/water/triisopropylsilane (95/2.5/2.5) to the cellulose-peptide conjugates for three hours, whereupon it was diluted with water (1:10) and submitted for ESI-LC/MS. The intact sequence of the peptide component of the cellulose-peptide conjugate (COOH-Glycine-NH2-Alanine-Proline-Ala-7-amino-4-methylcoumarin) was confirmed for its molecular weight.

### 4.3. Response and Sensitivity Assay

The fluorescence response and sensitivity assay of the cellulosic and nanocellulosic materials employed was previously outlined by Fontenot et al. [10]. Briefly, duplicates of ~2 mg of each biosensor were placed into a 96 well plate and 100 μL of a phosphate buffer solution (PBS) was added. A standard curve of the tripeptide substrate (1 to 0.0156 μmol/mL) was added to the 96 well plate with a total volume of 100 μL. One well containing only PBS was also included in the standard curve. To start the reaction for the response assay, 50 μL human neutrophil elastase (HNE) was added to the wells containing the biosensors and the standard curve for a total volume of 150 μL. For the sensitivity assay, 50 μL of HNE ranging from 2 to 0.0156 U/mL was added to the standard curve and to the biosensors to provide a total volume of 150 μL, from which the lowest sensitivity detection limit was determined. Fluorescent measurements commenced at 37 °C for 1 hour and measured at 1-min intervals while shaking for 3 s prior to measurement using a Biotech Synergy HT with a tungsten halogen lamp and photomultiplier detection (Winooski, VT, USA). The fluorescence measurements were acquired at 360 nm excitation and 460 nm emission. Fluorescent raw data were processed in Microsoft Excel 2013. The wound like fluid herein comprises of HNE in PBS. 

### 4.4. Surface Charge 

The investigation of the surface charge (zeta potential, ζ) values of the cellulose filter paper (CFP) was performed using an Anton Paar Surpass (Ashland, VA, USA). The electrophoretic mobility of the samples was measured using a cylindrical cell. All measurements were performed with a 1 mM KCl solution in deionized water at 21–23 °C. A 0.1M NaOH solution was used to adjust the KCl solution to pH 10.3 for FP. The following parameters were used for titration on the Anton Paar Surpass: aqueous solution of 0.100 M HCl, desired pH increment difference 0.200, volume increments 0.020 mL, pH minimum 2.5, and the pH maximum were slightly higher than the pH of the adjusted KCl solution. The flow rate ranged between 50 and 150 mL/min with a maximum pressure of 300 mbar (for the pressure and rinse program).

### 4.5. Kinetics Assay

The protocol of the fluorescence response assay was used for the fluorogenic kinetics assay; however, the biosensor concentration varied in duplicate concentrations between 0.5 and 4 mg (dissolved in 100 μL PBS). To start the reaction, 50 μL of 0.5 U/mL of human neutrophil elastase was added to both the free peptide substrate solution used to prepare the standard curve and for the biosensors, providing a total volume of 150 μL. 

Fluorescent measurements were conducted as described for the response and sensitivity assay, however, at 20 s intervals for a period of 10 min. The data were imported into GraphPad Prism software (6, GraphPad La Jolla, CA, USA) and analyzed as follows: create XY columns for sample data with enzyme kinetics, non-linear regression for curve fit, enzyme kinetics-substrate vs. velocity, and Michaelis–Menten to determine *K*_m_ and V_max_ values.

### 4.6. Circular Dichroism 

Circular dichroism (CD) studies were performed using a Jasco J-815 spectrometer with Spectra Manager 2 Software suite (Jasco, Easton, MD, USA). The CD measurements were carried out using 1 mm path length quartz cell. Peptide solutions were prepared at various concentrations in pH 7.4 PBS (0.5 M NaCl and 0.1 M NaH_2_PO_4_) containing 10% triflouroethanol (TFE). All spectra correspond to an average of three measurements, which were corrected using the baseline obtained for peptide free solutions. All data were converted to molar ellipticity and smoothed using a binomial function.

### 4.7. X-ray Diffraction (XRD)

All samples analyzed in the laboratory of Prof. John Wiley at the University of New Orleans Chemistry Department. The CFP, CNC, and NA samples were pressed into pellets and scanned in the θ–2θ reflection mode with a Philips X’pert powder diffractometer using Cu K radiation and a graphite monochromator. The data were obtained in a 2θ scale from 5° and 40° with a step size of 0.05 at 2.5 s per step with an overall run time of 30 min. No background corrections were made. The crystallinity indices (CrI) of the matrices were determined by subtracting the minimum intensity near 18° 2-θ (I_AM_) from the maximum intensity (at 22.625°–725° 2-θ, I_200_) and dividing the difference by the maximum intensity (I_200_) (Equation (1)) [30]. The crystallite size was calculated from the XRD patterns using the Scherrer formula [31] (Equation (2)). The terms of Equation (2) are shape factor (K) of 1, copper K radiation average wavelength (λ) is 1.5418 Ǻ, the full width at half maximum of the (200) peak (β) in radians, and the peak position divided by 2 of the (200) peak (θ) [32]. The crystallite models were drawn using Mercury software 3.5.1 (The Cambridge Crystallographic Data Centre Cambridge, UK), dividing the crystallite sizes from the Scherer equation calculations by the d_200_ interplanar spacing to get the number of chain layers. The tripeptide cellulose analogs were optimized using Chem3DPro 13.0 MM2 minimization energy calculations.

(1) $$CrI=\frac{I200-IAM}{I200}\times 100$$

(2) $$t=\frac{K*\lambda }{\beta *COS\left(\theta \right)}$$

### 4.8. Rietveld Refinement Method and Crystallite Models from the NA Diffraction Pattern

The Rietveld method was only used to refine the NA diffraction patterns in order to determine a crystallite size. The standard model diffraction patterns for cellulose I and cellulose II (cif) were based on work by Nishiyama et al. and more recently by French et al. [28,33]. Materials Analysis Using diffraction (Maud) 2.55 software [34] was used to apply the Rietveld method in order to determine the calculated diffraction patterns based on cellulose I, cellulose II, and amorphous patterns in order to resolve the broad overlapping cellulose I and cellulose II peaks and determine the crystallite size. Several parameter were modified in the Maud software during the refining process for the NA: the scale factor, quadratic background, and unit cell length a (layer I: the phase atom); cellulose I β: cell length a, scale factor, crystal size, and cell angle gamma (March-Dollase model: background and crystal size); and cellulose II: cell length a, scale factor, crystal size, and cell angle gamma (March-Dollase model: utilizing background and crystal size). 

### 4.9. Molecular Modeling Studies

Computational studies minimized the energy of the tripeptide substrate anchored on the five-chain 110 or 1–10 surfaces of cellulose in the following rotamer positions: (A) gauche-gauche; (B) gauche-trans; and (C) trans-gauche [11]. The peptide substrate models were built using GaussView 5.0.9 and optimized using a semiempirical Hamiltonian method (PM3) [35] contained in Gaussian 09 revision A.02 molecular orbital software (Gaussian inc., Wallingford, CT, USA).

## 5. Conclusions

We evaluated cotton-based cellulosic filter paper, nanocellulosic crystals, and nanocellulosic aerogel transducers conjugated to a tripeptide substrate using: (i) X-ray diffraction to determine their diffraction pattern, cellulose orientation, crystallite size, and crystallite model; (ii) Circular dichroism for assessment of the tripeptide substrate conformation, which revealed a β-turn confirmation; (iii) the bioactivity properties of the sensors via fluorescence response and kinetic properties that indicated the sensors provide a faster response to human neutrophil elastase compared to the free tripeptide substrate; and (iv) computational assessment of the cellulose tripeptide substrate analog in different orientations. Therefore, these materials, in particular, the nanocellulosic aerogel, have promising sensitivity, selectivity, and a faster kinetics response for detecting human neutrophil elastase, and are a promising sensor interface for a multilayered chronic wound dressing motif. Furthermore, the nanocellulosic sensors are adaptable for the detection of other biomarkers of clinical interest as well, via substitution of the biomolecule.

## Figures and Tables

**Table 1:** The crystallinity index values, crystallite size values, and crystallite model for the cellulosic filter paper (CFP), cotton nanocrystalline cellulose (CNC), and nanocellulosic aerogel (NA) transducer.

Name	Segal CrI (%)	Crystallite Size (Å)	Crystallite Model	Layers Molecules	Surface Charge (mV)
CFP	87	69.3	18	144	−10.2
NC	79	54.3	14	98	−68
NA I_β_	-	26	6	24	−19
NA II	-	65	16	122	−19
NA amorphous	-	19	5	24	−19

**Table 2:** Kinetic parameters for human neutrophil elastase (HNE) catalyzed hydrolysis of the tripeptide in solution and on the sensor.

Name	*k*_cat_ (s^−1^)	*K*_m_ (μM)	*k*_cat_/*K*_m_ (M^−1^·s^−1^)	V_max_ (s^−1^)	Corr. Coeff. (Correlation Coefficient)
Suc-APA-AMC	2.56	781.4	3272.33	2.17	0.9515
pCFP	0.1201	2.150	5,5860.5	0.1021	0.8645
pNA	1.67	202	8267.33	1.42	0.8935
pCNC	0.7732	23.07	3,3515.39	0.6572	0.9956

**Table 3:** Distance of AMC (7-amino-4-methylcoumarin) tripeptide substrate from the five-chain 110 or 1–10 surface of the cellulose floor.

Name	Distance from AMC to Cellulose Floor (A)
110 gg tripeptide	10.7
110 gt tripeptide	22.8
110 tg tripeptide	18.4
1 m 10 gg tripeptide	23.2
1 m 10 gt tripeptide	12.2
1 m10 tg tripeptide	9.9

## Abbreviations

AMC	7-amino-4-methylcoumarin
HNE	human neutrophil elastase
CFP	(cellulosic filter paper)
NA	nanocellulosic aerogel
CNC	cotton nanocrystalline cellulose
pCFP	peptide-cellulose conjugate on filter paper
pNA	peptide-cellulose conjugate on NA
pCNC	peptide-cellulose conjugate on NC
*k*_cat_	enzyme turnover rate
*K*_m_	enzyme binding affinity constant
g	gauche conformation
t	*trans* conformation
MAUD	materials analysis using diffraction
